# Expanding the Phenotype of Congenital Glucocorticoid Deficiency: An Iranian Patient with Cholestasis due to Pathogenic Variants in the *MC2R* Gene

**DOI:** 10.1155/2024/3201949

**Published:** 2024-08-05

**Authors:** Shohreh Maleknejad, Setila Dalili, Ameneh Sharifi, Afagh Hassanzadeh Rad, Reza Bayat, Bahareh Rabbani, Nejat Mahdieh

**Affiliations:** ^1^ Pediatric Diseases Research Center Guilan University of Medical Sciences, Rasht, Iran; ^2^ Growth and Development Research Center Tehran University of Medical Sciences, Tehran, Iran; ^3^ Cardiogenetic Research Center Rajaie Cardiovascular Medical and Research Institute Iran University of Medical Sciences, Tehran, Iran

## Abstract

Familial glucocorticoid deficiency is caused by variants in the *MC2R* and *MRAP* genes. We report an Iranian patient with congenital glucocorticoid deficiency and cholestasis due to pathogenic variants in the *MC2R* gene. This is the first documented case of a patient with conditions. Clinical evaluations and lab assessments were conducted on a six-month-old male infant. Next-generation sequencing identified the genetic causes of the disease, and Sanger sequencing confirmed the variants through segregation analysis. The clinical presentation included prolonged jaundice, progressive skin hyperpigmentation, seizures, fever, and a large umbilical hernia. Two variants in the *MC2R* gene, c.560delT and c.676G > *C*, were detected and classified as pathogenic and likely pathogenic, respectively. The cooccurrence of cholestasis and glucocorticoid deficiency illustrates the clinical heterogeneity caused by *MC2R* variants. The prevalence of c.560delT and c.676G > *C* between Iranian populations suggests these variants may be common. The high frequency of c.560delT could be attributed to a founder effect.

## 1. Introduction

Familial glucocorticoid deficiency (FGD), also known as hereditary unresponsiveness to adrenocorticotropic hormone (ACTH), is a heterogeneous autosomal recessive disorder. Mutations in *MC2R* and *MRAP* have been identified as causal factors for FGD. Notably, pure glucocorticoid deficiency adversely affects hepatocellular function, potentially leading to disruptions in bile acid and bilirubin synthesis and excretion [1]. In adrenalectomized rodents, studies have demonstrated bile stasis attributable to diminished bile turnover [2, 3]. Cholestasis is infrequently associated with adrenal insufficiency. Previous reports have documented isolated cortisol deficiency [4, 5] and congenital hypopituitarism [6] associated with cholestasis with or without elevation of gamma-glutamyl transferase [4–7]. There is also a report that severe cortisol deficiency in early infancy causes cholestatic hepatitis [6]. Moreover, evidence suggests a connection between neonatal liver failure and congenital adrenal hyporesponsiveness, as well as the occurrence of addisonian-like crises in congenital hypopituitarism and cholestatic jaundice [8, 9]. Conversely, alterations in bile flow have also been observed in adrenalectomized mice [2, 10]. Based on the aforementioned documents, it is prudent to incorporate noninvasive hormonal tests into the initial assessment before considering a liver biopsy in neonates and infants with cholestasis and hypoglycemia. This study documents a case of neonatal cholestasis and hepatosplenomegaly accompanied by seizure and hypoglycemia attributed to severe cortisol deficiency. While some authors have reported an association between cortisol deficiency and cholestasis, as well as the successful treatment of cholestasis with hydrocortisone replacement therapy, it is essential to underscore the importance of initially measuring cortisol levels in the evaluation of cholestasis, a condition rarely associated with adrenal insufficiency [4–7].

## 2. Materials and Methods

### 2.1. Genetic Investigations

Peripheral blood was taken for DNA extraction. WES was performed by an Illumina HiSeq 4000 platform with the mean read depth of 150X. The whole-exome sequencing raw data (FASTQ file) were analyzed using a core i7 CPU (central processor unit), 16 GB RAM in the Linux Ubuntu 20.04 operation system; briefly, FastQC tool (version 0.11.9) was applied for quality control of the reads. The adapters were removed using the Trimmomatic software (version 0.36).

The sequence reads were aligned to the reference genome of human (GRCh38/hg38) using Bowtie2 (Version 2.4.0) aligning tool generating SAM (Sequence Alignment/Map) file format. The SAM file was converted to BAM (Binary Alignment Map v.0.7.12) file by utilizing Picard. Local realignment of insertion/deletion (indels) was performed using the genome analysis toolkit (GATK v3.8). Minor insertions/deletions (indels) and single-nucleotide variations (SNVs) were selected and filtered based on quality metrics, including variant quality score recalibration (VQSR). The Ensemble Variant Effect Predictor (VEP) tool was used to annotate the resulting VCF (Variant Call Format) file. A comprehensive functional annotation was performed using databases such as dbSNP, ClinVar, and the Exome Aggregation Consortium (ExAC, https://exomad.broadinstitute.org/). A minimal allele frequency (MAF) of more than 0.01 in the ExAC and Exome Sequencing Project 6500 (https://evs.gs.washington.edu/EVS/) was considered to filter the variants. The variants were named according to the HGVS (https://hgvs-nomenclature.org/) recommendations and were classified using ACMG 2015. Available bioinformatics tools including MutationTaster (https://www.mutationtaster.org/), SIFT (https://sift.bii.a-star.edu.sg/), PROVEAN (https://provean.jcvi.org/index.php), and CADD-splice (https://cadd.gs.washington.edu/home) were used to predict the pathogenicity of variants. The identified pathogenic variants were confirmed using Sanger sequencing of the given regions in the *MC2R* gene (NM_000529.2).

### 2.2. Review Literature

We conducted a comprehensive analysis of individual data, integrating clinical characteristics and genetic analyses to gain insight into the disease caused by two variants. Our investigation into the genetics of familial glucocorticoid deficiency (FGD) involved an extensive search of databases including PubMed, ScienceDirect, and John Wiley and Springer. These databases yielded relevant information. We employed keywords related to FGD, such as “variant,” “c.560delT,” “c.676G > C,” “FGD,” “p.Val187Alafs^*∗*^29, ” “p.Gly226Arg,” and “*MC2R*,” along with the term “phenotypic variability” to ensure inclusivity. The collected data encompassed patient demographics, including numbers, age of onset, gender distribution, clinical symptoms, variant types, genotypic profiles, and clinical test outcomes. To identify the most prevalent variants, we applied criteria from relevant studies to select those with the highest frequency. Subsequently, we calculated the frequencies of deletion and missense mutations across diverse patient cohorts and geographical regions.

### 2.3. Clinical Features

**Figure 1:**
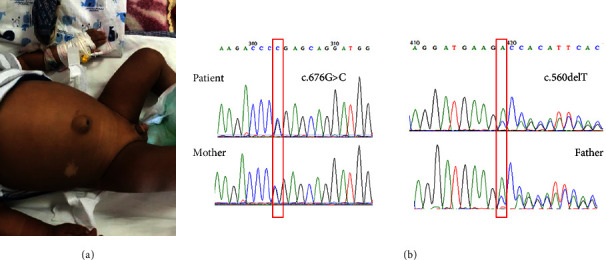
(a) Skin hyperpigmentation and umbilical hernia. (b) Electropherogram of the patient and his parents.

A six-month-old male infant, who had been admitted twice due to prolonged jaundice and progressive skin hyperpigmentation since birth, was brought to the hospital after experiencing a seizure with a fever on the first day of life. He was the first child of unrelated parents, was formula-fed, and displayed average height and weight (7.800 kg and 67 cm). During the physical examination, generalized jaundice and hyperpigmentation were noted. The liver and spleen were palpable 4 cm and 3 cm below the costal margin, respectively. Additionally, he presented with a sizable umbilical hernia measuring 3 cm (Figure 1). Routine chest examination, including heart assessment, was conducted. Generalized hyperpigmentation was observed along with a penile stretch length of 2.5 cm and palpable testes in the scrotum. The eye examination yielded normal results.

## 3. Results

The patient's laboratory findings are detailed in Table 1. Thyroid hormone levels were within normal range. The laboratory results of the patient are detailed in Table 1. Thyroid hormone levels were within normal range. Comprehensive metabolic screening for inherited metabolic disorders, which included tandem mass spectrometry (MS), urinary organic acid profile, NH3 levels, and long-chain panel levels, all yielded normal results. Tests for hepatitis, toxoplasmosis, rubella, cytomegalovirus, and herpes simplex returned negative results. Despite elevated ACTH levels in the absence of mineralocorticoid deficiency, low plasma cortisol levels indicated a diagnosis of isolated glucocorticoid deficiency. This led to the initiation of hydrocortisone replacement therapy. After one month, bilirubin levels normalized; after four months, all liver function tests returned to normal. Throughout the eight-month follow-up period, ACTH levels remained low despite hydrocortisone replacement therapy being administered and normal mineralocorticoid function observed. No hypoglycemic episodes were reported during this monitoring period.

Two known genetic variations in the *MC2R* gene (NM_000529.2), specifically c.560delT and c.676G > C, were detected. These variants were determined to be pathogenic and likely pathogenic, respectively.

### 3.1. Clinical Features of Patients with c.560delT

**Figure 2:**
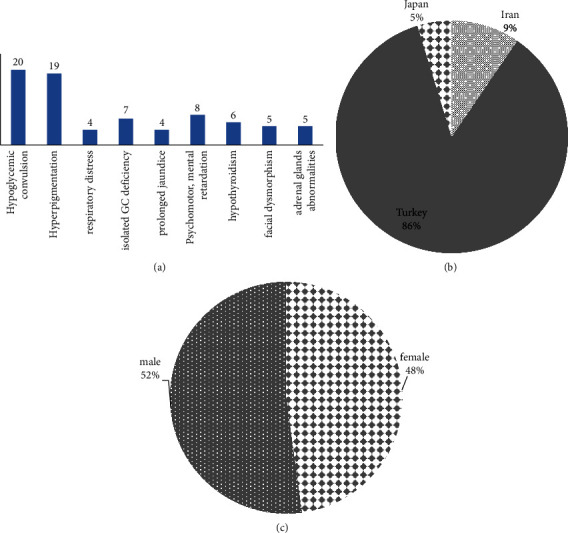
(a). The number of affected people according to the phenotypes they have shown. (b) Percentage of patients with MC2R in different countries. (c) The number of study cases separated based on their gender.

Twenty-one cases were identified to carry the c.560delT mutation, with 18 patients exhibiting a homozygous mutation one patient displaying a compound heterozygous genotype. The age of symptom onset was recorded for a total of 23 patients from 21 unrelated families, with an average age of onset at 3.8 months. There were 11 male and 10 female patients, while gender information was unavailable for 2 cases (Figure 2(c)). Hyperpigmentation, hypoglycemia, respiratory distress, and hypothyroidism were found in 20, 16, 4, and 4 cases, respectively (Figure 2(a)). Out of 21 patients, 6 cases experienced isolated GC deficiency, 3 from mild facial dimorphism, and two from ADHD. In the biochemical test, 9 cases had elevated serum Na and K. The plasma ACTH level was high in 5 cases, while cortisol levels were low. Tall stature was observed in 11 cases (Table 2).

### 3.2. Clinical Features of Patients with c.676G > C

Among a group of 23 patients, 2 individuals exhibited homozygosity, while an additional two displayed compound heterozygosity for the c.676C > G variant. These patients presented with similar clinical manifestations, including hypoglycemia, hypothyroidism, and reduced levels of sodium (NA), potassium (K), and cortisol. Notably, their adrenocorticotropic hormone (ACTH) levels were elevated compared to the normal.

### 3.3. Distribution of the Variants

**Figure 3:**
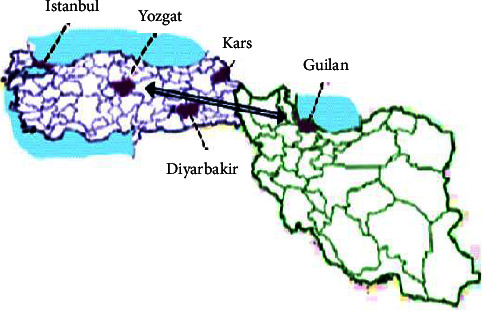
Geographical distribution of c.560delT and c.676C > G mutations identified in this study. The first report of the c.560delT mutation was in a family from Iran who was in a family with two siblings who originated from Northern Iran, close to the Turkish border. The c.560delT variant showed a wider distribution most likely reflecting migration from the East to the West of Turkey and has been reported in previous article.

A globally representative cohort of nearly 23 patients diagnosed with fibroglandular dysplasia (FGD) was assembled from various scholarly articles. The findings revealed a notable prevalence of homozygosity, observed in 16 out of 21 families. The research highlighted that 87.5% of the mutations were identified as c.560delT, while missense variants (c.676C > G) accounted for 16%. The distribution of these variants was graphically illustrated (see Figure 2(b)). The c.560delT mutation predominantly manifested in patients from Turkey and one family from Northern Iran, with eighteen families hailing from Turkey (Figure 3).

## 4. Discussion

Familial glucocorticoid deficiency (FGD) results from a resistance to the action of adrenocorticotropic hormone (ACTH) on the adrenal cortex, leading to an isolated deficiency in glucocorticoids. This condition manifests as an isolated deficiency in glucocorticoids. FGD is a rare autosomal recessive disorder that typically presents in infancy or early childhood. Clinical features include hyperpigmentation, recurrent infections, failure to thrive, hypoglycemic episodes, and seizures, which can progress to coma or death. Diagnostic evaluation reveals decreased levels of cortisol and androgens along with elevated ACTH levels, while the renin-aldosterone axis remains unaffected. [6]. This genetically heterogeneous disorder usually results from variants in the *MC2R* gene (FGD type 1) and *MRAP* gene (FGD type 2), though WES has identified variants in other genes causing other types of FGD [12, 17]. This study details the clinical manifestations of 23 patients (from 21 distinct families) harboring mutations c.560delT (p.Val187Alafs^*∗*^29) and c.676G > C (p.Gly226Arg) and analyzed the geographical prevalence of these variants. Furthermore, a novel case is presented involving a family with cholestasis resulting from these deleterious mutations in the *MC2R* gene is presented, contributing to the broadening of the phenotypic spectrum associated with congenital glucocorticoid deficiency.

To the best of our knowledge, only a 32-day-old male infant has been reported with infantile cholestasis due to a homozygous variant c.763_764delAT (p.Met255Valfs^*∗*^17) in the *MC2R* gene [18]. In this study, we present a novel Iranian case exhibiting unique clinical manifestations of FGD1. The patient was found to be compound heterozygous for two previously reported variants in the *MC2R* gene: c.560delT (p.Val187Alafs^*∗*^29) [19] as a pathogenic variant and c.676G > C (p.Gly226Arg) [12] classified as a likely pathogenic variant. This compound heterozygosity resulted in a distinct phenotype previously unreported in the literature.

*MC2R* expressed in the zona fasciculata encodes the ACTH receptor, and the *MRAP* gene encodes an accessory protein of MC2R (melanocortin−2 receptor accessory protein). Pathogenic variants of the *MC2R* gene that produce an aberrant protein can downregulate the cortisol synthesis pathway and abolish ACTH stimulation; defects in this pathway could lead to various clinical manifestations of FGD, including hyperpigmentation, hypoglycemia, eczema, failure to thrive, jaundice, and increased susceptibility to infection [20–22].

In addition to high levels of ACTH, 79% of *MC2R*-related patients have hyperpigmentation; hypoglycemia is also observed in 77% of patients, and seizure, jaundice, and vomiting are documented in 20%, 30%, and 14% of the affected individuals, respectively [12]. Respiratory stress and recurrent infection are also seen around 10% of patients [12]; almost all these clinical manifestations were noted. Malnutrition has also been reported in some cases which was not present in our patient. An accidental finding in our case, an umbilical hernia was documented which has not been reported to the best of our knowledge [23]. In our study, 18 patients had a homozygous mutation, while one patient had a compound heterozygous genotype. The mean age of onset was identified to be 3.8 months. Hyperpigmentation and hypoglycemia are the most common symptoms associated with this variant (Figure 2(a)).

It is important to consider that the frequency of certain variants may be attributed to a founder effect, while others may arise as mutational hotspots [24]. Additionally, some variants may exhibit ethnicity-specific distributions due to founder effects. For instance, specific variants in genes such as GJB2 variants and CYP21A2 have been found to have elevated frequencies in particular populations [25, 26]; for example, the c.560delT is variant is prevalent among Turkish patients [12]. This variant may have a wider distribution in this country, and it is common likely due to migration from the east to west of Turkey. Notably, the c.560delT mutation was initially identified in an Iranian family with Northern Iranian ancestry, suggesting a potential founder effect within the Iranian population [11]. Furthermore, the c.560delT and c.676G > C variants have been reported in Turkish populations, indicating a shared genetic landscape between these populations [12, 14].

In conclusion, when evaluating FGD patients, an initial measurement of cortisol levels is recommended for the initial assessment of cholestasis.

## 5. Conclusion

The findings from this study, combined with those from other research focusing on specific mutations (c.560delT (p.Val187Alafs^*∗*^29) (19) and c.676G > C) in *MC2R*, indicate that these patients with these mutations commonly exhibit clinical symptoms such as hyperpigmentation, frequent infections, lack of growth, hypoglycemic attacks, and seizures, which can potentially progress to coma or result in fatality.

## Figures and Tables

**Table 1:** Laboratory results of the studied patient.

Lab test	On admission					Reference range, children
	This patient	Iranian P1F1	Iranian P2F1	Turkish P1 F2	Turkish P1 F11	
Na	136	136	129	129	141	135–145 mEq/l
K	4	3,9	4.0	7	4.1	3–5 meq/l
Cl	104	—	—	—	—	96–106 meq/l
ALT	141U	—				0–41 U/L
AST	574	—				0–37 U/L
GGT	41	—				11–49 U/L
LDH	690	—				240–480 U/L
ALP	940	—				250–1200 U/L
Bilirubin	8.9	—				0.1–1.2 mg/dl
Conjugated bilirubin	3.4	—				<0.3 mg/dl
Ca	8.8	—				8–10 mg/l
P	4.8	—				3–7 mg/l
FBS	2.9	—				8.0–10.0 mg/dl
ACTH	4970	—		1643		9–46 pg/ml
Cortisol	<1	0.5	1.0		2.48	5–15 mcg/dl
Insulin	1	—				Up to 5 *µ*U/ml

**Table 2:** Information and data related to all patients who carried c.560delT and c.676C > G variants in all articles and in the study.

Family	No	Age	Consanguinity	Sex	Ethnicity	Genotype	Hypoglycemic convulsion	Hyperpigmentation	Respiratory distress	Isolated GC deficiency	Prolonged jaundice	Psychomotor, mental retardation	Hypothyroidism	Facial dysmorphism	Adrenal glands abnormalities	Symptoms	Ref
1	1	19M	N	M	Iran	c.437G > A/c.560delT	Hypoglycemia	Pigmentation									[11]
	2	12M	N	F		c.437G > A/c.560delT	Hypoglycemia	Pigmentation						Viral illness			[11]

2	1	2W	N	M	Turkey	c.560delT/c.560delT		Hyperpigmentation	Respiratory distress		Prolonged jaundice		Mild facial dysmorphism	Unilateral renal hypoplasia	Pneumonia, recurrent iron deficiency anemia	Tall stature	[12]

3	1	1W	N	F	Turkey	c.560delT/c.560delT	Hypoglycemic	Hyperpigmentation	Isolated GC deficiency	Prolonged jaundice	Subclinical hypothyroidism					Tall stature	[12]

4	1	2W	N	M	Turkey	c.560delT/c.560delT	Hypoglycemic	Hyperpigmentation	Respiratory distress						Bilateral hypoplastic adrenal glands	Secundum type atrial septal defect, Severe hyponatremia, hypoplasia of corpus callosum	[12]
	1	3W	N	F		c.560delT/c.560delT	Hypoglycemic	Hyperpigmentation	Isolated GC deficiency	Subclinical hypothyroidism	Mild hyponatremia						[12]

5	1	1W	N	F	Turkey	c.560delT/c.560delT	Hypoglycemic	Hyperpigmentation		Isolated GC deficiency	Moderate mental retardation	Subclinical hypothyroidism	Mild facial dysmorphism, laterally sparse eye lashes)	Bilateral hypoplastic adrenal	Mild ptosis		[12]

6	1	1W	N	F	Turkey	c.560delT/c.560delT	Hyperpigmentation		Isolated GC deficiency	Prolonged jaundice	Moderate learning difficulty	Normal adrenal USG					[12]

7	1	5W	Y	M	Turkey	c.560delT/c.560delT	Hypospadias	Hyperpigmentation	Neonatal respiratory distress		Persistently Mild learning difficulty	Hyponatremia, unilateral cryptorchidism				Tall stature, delayed myelinization	[12]

8	1	1W	N	M	Turkey	c.560delT/c.560delT	Hypoglycemic convulsion	Hyperpigmentation	Isolated GC deficiency	Prolonged jaundice difficulty	ADHD, mild learning	Transient subclinical hypothyroidism	Brachycephaly				[12]

9	1	1W	N	M	Turkey	c.560delT/c.560delT	Hyperpigmentation		Isolated GC deficiency		ADHD, mild learning difficulty	Mild facial dysmorphism	Mild ptosis	Hypoglycemia			[12]

10	1	1W	N	M	Turkey	c.560delT/c.560delT	Hypoglycemic convulsion	Hyperpigmentation		Isolated GC deficiency				Vomiting		Normal adrenal	[12]

11	1	42W	Y	F	Turkey	c.560delT/c.560delT	Normal appearance	Hyperpigmentation						Congenital primary hypothyroidism	Hypoplastic adrenal glands	Salt-wasting crises, pulmonary hypertension	[13]

12	1	NK	Y	NK	Turkey	c.676C > G/c.676C > G	Hypoglycemia						Hypothyroidism				[12]

13	1	5M	N	F	Turkey	c.560delT/c.560delT	Hypoglycemia	Hyperpigmentation					Hypotonia				[14]

14	1	12M	N	F	Turkey	c.560delT/c.560delT	Hypoglycemia	Hyperpigmentation			Psychomotor retardation						[14]

15	1	18M	Y	F	Turkey	c.676C > G/c.676C > G						Psychomotor retardation	Congenital hypothyroidism	Recurrent epileptic seizures			[14]

16	1	5D	N	F	Turkey	c.560delT/c.560delT	Hypoglycemia		Respiratory distress								[14]

17	1	2D	N	M	Japan	c.307G > A/c.676C > G	Hypoglycemia	Hyperpigmentation					Poor weight gain	Seizures			[15]

18	1	1 D-11M	N	M	Turkey	c.560delT/c.560delT	Hypoglycemic	Hyperpigmentation							Hypoglycemia		[16]

19	1	1 D-11M	N	M	Turkey	c.560delT/c.560delT	Hypoglycemic	Hyperpigmentation							Hypoglycemia		[16]

20	1	1 D-11M	N	M	Turkey	c.560delT/c.560delT	Hypoglycemic	Hyperpigmentation							Hypoglycemia		[16]
	2	1 D-11M	N	F		c.560delT/c.560delT	Hypoglycemic	Hyperpigmentation							Hypoglycemia		[16]

21	1	1 D-11M	N	M	Iran	c.560delT/c.676G > C		Hyperpigmentation							Seizure		This study

W: week, M: month, NK: unreported, N: no, Y: yes, P: patient, F: family.

## Data Availability

There are no other data available. The sequence data of the patient were submitted to the SRA database with a submission number of SRA: SUB14289426.

## References

[1] Lane E., Murray K. F. (2017). Neonatal cholestasis. *Pediatric Clinics of North America*.

[2] Quinn M., Ueno Y., Pae H. Y. (2012). Suppression of the HPA axis during extrahepatic biliary obstruction induces cholangiocyte proliferation in the rat. *American Journal of Physiology - Gastrointestinal and Liver Physiology*.

[3] Lee W.-C., Shih S.-C., Wang H.-Y., Wu C.-L. L. S.-Y., Lee S. Y., Ku H. C. (2018). Adrenal insufficiency associated with cholestatic jaundice: a case report. *International Journal of Gerontology*.

[4] Al-Hussaini A., Almutairi A., Mursi A., Alghofely M., Asery A. (2012). Isolated cortisol deficiency: a rare cause of neonatal cholestasis. *Saudi Journal of Gastroenterology*.

[5] Chan U., Chan W. T., Ting W. H., Ho C. S., Liu H. C., Lee H. C. (2017). Cholestasis caused by panhypopituitarism and acquired cytomegalovirus infection in a 2-month-old male infant: a case report. *Medicine*.

[6] Gonc E. N., Kandemir N., Andiran N., Ozon A., Yordam N. (2006). Cholestatic hepatitis as a result of severe cortisol deficiency in early infancy: report of two cases and review of literature. *Turkish Journal of Pediatrics*.

[7] Di Dato F., Capalbo D., Mirra R., Del Vecchio Blanco F., Salerno M., Iorio R. (2021). Case report: neonatal cholestasis as early manifestation of primary adrenal insufficiency. *Frontiers in pediatrics*.

[8] Cheung M., Bansal S., Aw M. M., Buchanan C. R., Mieli-Vergani G., Dhawan A. (2003). Liver failure in a neonate with congenital adrenal hyporesponsiveness. *European Journal of Pediatrics*.

[9] Lee W. S., Lum L. C., Harun F. (2003). Addisonian-like crisis in congenital hypopituitarism and cholestatic jaundice. *Medical Journal of Malaysia*.

[10] Telkka A., Kuusisto A. N. (1962). Bile flow in adrenalectomized rats. *Acta Endocrinologica*.

[11] Lin L., Hindmarsh P. C., Metherell L. A. (2007). Severe loss-of-function mutations in the adrenocorticotropin receptor (ACTHR, MC2R) can be found in patients diagnosed with salt-losing adrenal hypoplasia. *Clinical Endocrinology*.

[12] Guran T., Buonocore F., Saka N. (2016). Rare causes of primary adrenal insufficiency: genetic and clinical characterization of a large nationwide cohort. *Journal of Clinical Endocrinology and Metabolism*.

[13] Kardas Yildiz A., Bulbul A., Ozer Bekmez B. (2023). A rare presentation of homozygous pathogenic variant in MC2R gene with salt-wasting crisis in a neonate. *Molecular Syndromology*.

[14] Özbek M. N., Demiral M., Unal E., Karaşin N. D., Baran R. T., Demirbilek H. (2021). A rare and preventable aetiology of neurodevelopmental delay and epilepsy: familial glucocorticoid deficiency. *Journal of Pediatric Endocrinology & Metabolism*.

[15] Sasasaki T., Naoko A., Narumi S. (2015). A case of ACTH resistance with generalized hyperpigmentation at birth. *ESPE Abstracts*.

[16] Ozturan E. K., Bas F., Abali Z. Y. (2022). Evaluation of early puberty in patients with MC2R deficiency. *Hormone Research in Paediatrícs*.

[17] Akın L., Kurtoğlu S., Kendirci M., Akın M. A. (2010). Familial glucocorticoid deficiency type 2: a case report - case report. *Journal of clinical research in pediatric endocrinology*.

[18] Alsaedi A., Kamal N. M., Bakkar A., Althobaiti E., Naeem M., Kamal M. (2022). Novel melano-cortin-2-receptor gene mutation presenting with infantile cholestasis: a case report. *Clinical Medicine Insights: Case Reports*.

[19] Clark A. J., Metherell L. A., Cheetham M. E., Huebner A. (2005). Inherited ACTH insensitivity illuminates the mechanisms of ACTH action. *Trends in Endocrinology and Metabolism*.

[20] Chan L. F., Clark A. J., Metherell L. A. (2008). Familial glucocorticoid deficiency: advances in the molecular understanding of ACTH action. *Hormone Research in Paediatrícs*.

[21] Tunkara H., Topor L. S. (2022). 50 Years ago in TheJournalofPediatrics: isolated cortisol deficiency due to ACTH resistance: an early description of familial glucocorticoid deficiency. *The Journal of Pediatrics*.

[22] Akin L., Kurtoglu S., Kendirci M., Akin M. A. (2010). Familial glucocorticoid deficiency type 2: a case report - case report. *Journal of Clinical Research in Pediatric Endocrinology*.

[23] Novoselova T. V., Chan L. F., Clark A. J. L. (2018). Pathophysiology of melanocortin receptors and their accessory proteins. *Best Practice & Research Clinical Endocrinology & Metabolism*.

[24] Davoudi-Dehaghani E., Zeinali S., Mahdieh N., Shirkavand A., Bagherian H., Tabatabaiefar M. A. (2013). A transversion mutation in non-coding exon 3 of the TMC1 gene in two ethnically related Iranian deaf families from different geographical regions; evidence for founder effect. *International Journal of Pediatric Otorhinolaryngology*.

[25] Rabbani B., Mahdieh N., Haghi Ashtiani M. T., Akbari M.-T., Rabbani A. (2011). Molecular diagnosis of congenital adrenal hyperplasia in Iran: focusing on CYP21A2 gene. *Iranian Journal of Pediatrics (English edition)*.

[26] Mahdieh N., Mahmoudi H., Ahmadzadeh S., Bakhtiyari S. (2016). GJB2 mutations in deaf population of Ilam (Western Iran): a different pattern of mutation distribution. *European Archives of Oto-Rhino-Laryngology*.

